# Evaluating Whether Radiofrequency Irradiation Attenuated UV-B-Induced Skin Pigmentation by Increasing Melanosomal Autophagy and Decreasing Melanin Synthesis

**DOI:** 10.3390/ijms221910724

**Published:** 2021-10-03

**Authors:** Hyoung Moon Kim, Seyeon Oh, Jin Young Yang, Hye Jin Sun, Miran Jang, Donghwan Kang, Kuk Hui Son, Kyunghee Byun

**Affiliations:** 1 Department of Anatomy & Cell Biology, Gachon University College of Medicine, Incheon 21936, Korea; drmac12@me.com; 2 Functional Cellular Networks Laboratory, Lee Gil Ya Cancer and Diabetes Institute, Gachon University of Medicine, Incheon 21999, Korea; seyeon8965@gmail.com (S.O.); roswellgirl111@gmail.com (J.Y.Y.); 3 Jeisys Medical Inc., Seoul 08501, Korea; sunhj@jeisys.com (H.J.S.); lyla@jeisys.com (M.J.); kang@jeisys.com (D.K.); 4 Department of Thoracic and Cardiovascular Surgery, Gachon University Gil Medical Center, Gachon University, Incheon 21565, Korea

**Keywords:** melanosomal autophagy, autophagosome, radiofrequency microneedling, ultraviolet B, skin pigmentation

## Abstract

Autophagy is involved in the degradation of melanosomes and the determination of skin color. TLR4 and tumor necrosis factor (TNF) signaling upregulates NF-kB expression, which is involved in the upregulation of mTOR. The activation of mTOR by UV-B exposure results in decreased autophagy, whereas radiofrequency (RF) irradiation decreases TLR4 and TNF receptor (TNFR) expression. We evaluated whether RF decreased skin pigmentation by restoring autophagy by decreasing the expression of TLR4 or TNFR/NF-κB/mTOR in the UV-B-irradiated animal model. UV-B radiation induced the expressions of TNFR, TLR, and NF-κB in the skin, which were all decreased by RF irradiation. RF irradiation also decreased phosphorylated mTOR expression and upregulated autophagy initiation factors such as FIP200, ULK1, ULK2, ATG13, and ATG101 in the UV-B-irradiated skin. Beclin 1 expression and the expression ratio of LC3-I to LC3-II were increased by UV-B/RF irradiation. Furthermore, melanin-containing autophagosomes increased with RF irradiation. Fontana-Masson staining showed that the amount of melanin deposition in the skin was decreased by RF irradiation. This study showed that RF irradiation decreased skin pigmentation by restoring melanosomal autophagy, and that the possible signal pathways which modulate autophagy could be TLR4, TNFR, NF-κB, and mTOR.

## 1. Introduction

Melanin is a nitrogen-containing pigment made from the melanin precursor L-tyrosine and is deposited in melanosomes, which are subcellular lysosome-like organelles [1]. Both keratinocytes and melanocytes are involved in melanogenesis in the skin: melanocytes produce the melanin and deliver it to the keratinocytes for skin protection [2].

Human skin color is determined by the balance between melanin synthesis and degradation [3]. Microenvironment alterations caused by ultraviolet (UV) radiation, free radicals, and inflammation change this balance, and such changes eventually result in either skin depigmentation or hyperpigmentation [4,5].

Melanogenesis is initiated by UV via the melanocortin-1 receptor [6]. By binding the α-melanocyte-stimulating hormone (α-MSH) to MC1R, the melanocyte-inducing transcription factor (MITF) is activated and increases tyrosinase, which is a rate-limiting enzyme of melanogenesis [6].

Autophagy is a vital cellular catabolic system for maintaining tissue homeostasis that eliminates aggregated or misfolded proteins and dysfunctional organelles [7]. Autophagy is also involved in melanosome degradation. Melanosomes are degraded by autophagy after being transferred from melanocytes to keratinocytes [8]. Moreover, melanosomes accumulated in melanocytes due to disrupted transport are also degraded by autophagy [9].

Solar UV upregulates the mammalian target of the rapamycin complex (mTORC), which inhibits unc-51-like autophagy-inhibiting kinase (ULK) 1 and decreases the complex formation of ULK1 with autophagy-related protein (ATG) 13 and the 200 kDa family-interacting protein (FIP200), thereby decreasing autophagy [10]. 

The activation of nuclear factor kappa-light-chain-enhancer of activated B cells (NF-κB) as an upstream target of mTORC1 inhibits autophagy [11,12,13]. Furthermore, the canonical NF-κB pathway is activated by pro-inflammatory pathways like the tumor necrosis factor (TNF) receptors, Toll-like receptors (TLR), and antigen receptors [14,15]. TNF has been shown to induce NF-κB activation, thereby decreasing autophagy in various cancer cells such as Ewing’s sarcoma, breast, and leukemia cancer cell lines. Conversely, the downregulation of NF-κB reactivates the autophagy process [11,16]. TLR4, an upstream target of NF-κB, is also known to dysregulate mTORC-dependent autophagy [17]. 

UV-B increases the production of various inflammatory molecules such as interleukin (IL)-1α, IL-1β, IL-6, IL-8, and TNF-α in keratinocytes [18,19,20]. UV-B-irradiated keratinocytes also secrete high-mobility group box 1 (HMGB1), which is a ligand of TLR4 [21]. These proteins are associated with post-inflammatory hyperpigmentation, which results from the overproduction or irregular dispersion of melanin [22]. 

Radiofrequency (RF) irradiation has been shown to decrease IL-6, IL-8, and HMGB1 expression in UV-B-irradiated keratinocytes [21], as well as the expressions of NF-κB, TNF-α, and TLR4 in the UV-B-irradiated animal model, thereby decreasing keratinocyte proliferation and pigment accumulation [21]. RF could decrease skin pigmentation by modulating skin inflammation through the downregulation of various pro-inflammatory signals like NF-κB, TNF-α, and TLR4. 

It is well known that UV-B irradiation increases various inflammatory cytokines and increases NF-κB, which eventually decreases melanosomal autophagy and increases skin pigmentation. Even though it is known that RF could decrease skin pigmentation, research concerning the mechanism of how skin pigmentation could be decreased by RF has mainly been focused on modulating inflammation. We thought it could be possible that RF decreases skin pigmentation by decreasing skin inflammation, which eventually leads to an increase in melanosomal autophagy. We hypothesized that RF downregulates TNF-α and TLR4, thereby downregulating NF-κB, and decreasing mTOR expression to restore melanosomal autophagy. In this study, we investigated the effects of RF irradiation on autophagy and skin pigmentation in the UV-B radiation animal model.

## 2. Results

### 2.1. RF Decreased the Expressions of TNFR, TLR4, and NF-κB

**Figure 1:**
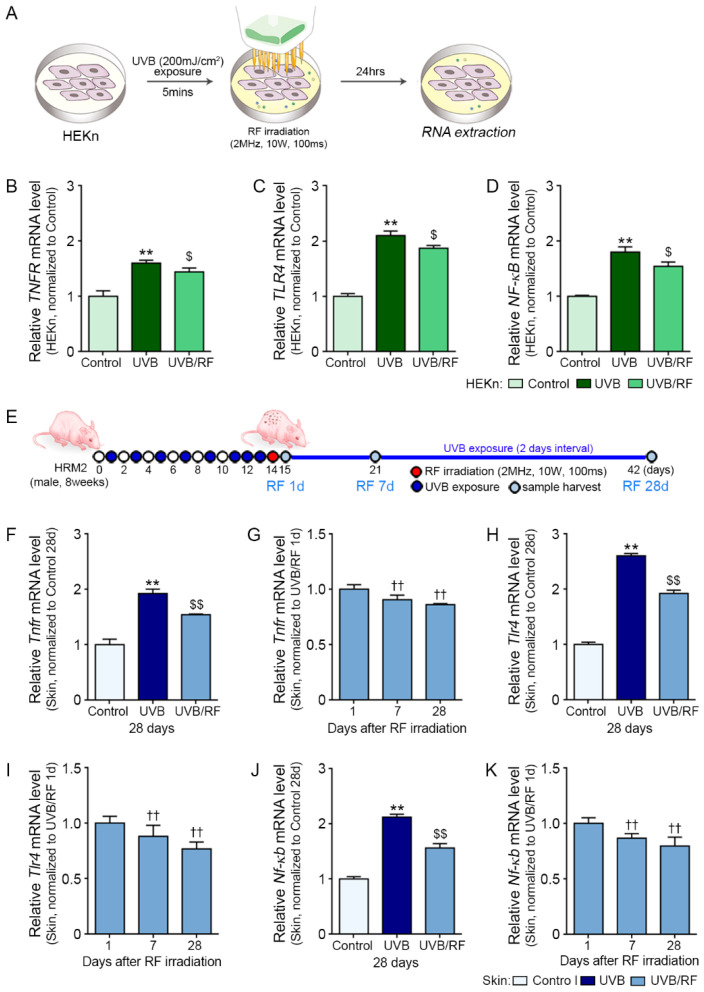
Downregulation of the expressions of TNFR, TLR4 and NF-κB by RF irradiation. (**A**) HEKn were irradiated either with UV-B (200 mJ/cm^2^) or with UV-B/RF (2 MHz, 10 W for 100 ms) in vitro, or otherwise not irradiated (control). The mRNA expression levels of (**B**) TNFR, (**C**) TLR4, and NF-κB (**D**) were determined in HEKn. The mRNA levels in HEKn were measured by qRT-PCR, normalized versus ACTB, and expressed relative to levels in the control group. (**E**) Mouse skins were irradiated either with UV-B (200 mJ/cm^2^) or with UV-B/RF (2 MHz, 10 W for 100 ms) in vivo, or otherwise not irradiated (control 28 days). The mRNA expression levels of (**F**,**G**) TNFR, (**H**,**I**) TLR4, and (**J**,**K**) NF-κb were determined in the skin tissue. The mRNA levels in the mouse skins were validated by qRT-PCR, normalized versus ACTB, and expressed relative to levels in the control 28 days group or 1 day after RF irradiation. Data are presented as mean ± SD. **, *p* < 0.01, vs. control (HEKn) or control 28 days (Skin); $, *p* < 0.05; $$, *p* < 0.01, vs. UV-B (HEKn) or UV-B 28 days (Skin); ††, *p* < 0.01, vs. 1 day after RF irradiation (Mann–Whitney U test). HEKn, human epidermal primary keratinocytes; NF-κB, nuclear factor kappa-light-chain-enhancer of activated B cells; RF, radiofrequency; TNFR, tumor necrosis factor receptor; TLR4, Toll-like receptor 4; UV-B, ultraviolet-B; UV-B/RF, UV-B plus RF.

First, we evaluated whether RF irradiation decreased the expressions of TNFR, TLR4, and NF-κB in the UV-B-irradiated human primary epidermal keratinocytes (HEKn cells). The expressions of TNFR, TLR4, and NF-κB were significantly increased by UV-B radiation in HEKn cells, whereas RF irradiation significantly decreased them at 24 h after RF irradiation (Figure 1A–D).

Statistical differences of all factors among the control, UV-B, and UV-B/RF groups were compared at the end of the experiment (28 days after RF irradiation). The changes of all factors by time points after RF irradiation were evaluated at 1, 7, and 28 days after RF irradiation, since we wanted to evaluate how long the RF effect continued. The statistical difference among time points was also compared (Figure 1E).

TNFR expression in UV-B-irradiated skin was significantly higher than those in the control and UV-B/RF groups. In the UV-B/RF group, TNFR expression was highest at 1 day after RF but decreased with time (Figure 1F,G).

The expressions of TLR4 and NF-κb were significantly increased by UV-B irradiation and were significantly higher than those in the control and UV-B/RF groups. In the UV-B/RF group, the expressions of TLR4 and NF-κb were highest at 1 day after RF irradiation but decreased with time (Figure 1H–K). 

### 2.2. RF Decreased mTOR Expression and Induced the Expression of Autophagy Initiation Factors in UV-B-Irradiated Skin

**Figure 2:**
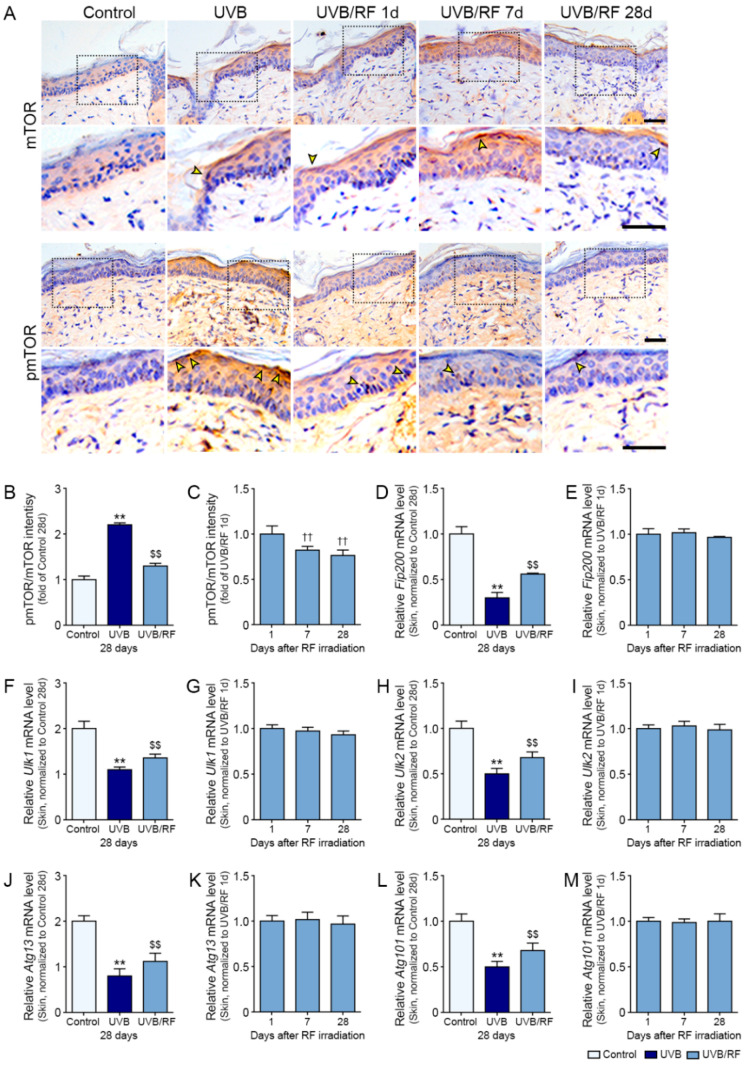
Activation effects of autophagy initiation response by RF irradiation in the UV-B-exposed animal model. (**A**) mTOR and pmTOR expression (arrows) in the epidermis of the UV-B-exposed mouse skins were assessed immunohistochemically (scale bar = 100 µm); (**B**,**C**) Quantitative ratios of representative mTOR and pmTOR images. Mouse skins were either with UV-B or with UV-B/RF in vivo, or otherwise not irradiated (control 28 days); (**D**–**M**) The mRNA expression levels of (**D**,**E**) FIP200, (**F**,**G**) ULK1, (**H**,**I**) ULK2, (**J**,**K**) ATG13, and (**L**,**M**) ATG101 were determined in autophagy initiation response-related factors in skin tissue. All mRNA levels were measured by qRT-PCR, normalized versus ACTB, and expressed relative to levels in the control 28 days group or 1 day after RF irradiation. Data are presented as mean ± SD. **, *p* < 0.01, vs. control 28 days; $$, *p* < 0.01, vs. UV-B 28 days; ††, *p* < 0.01, vs. 1 day after RF irradiation (Mann–Whitney U test). ATG, autophagy-related protein; mTOR, mechanistic target of rapamycin complex; pmTOR, phosphorylated mTOR; RF, radiofrequency; ULK, unc-51-like autophagy activating kinase 1; UV-B, ultraviolet-B; UV-B/RF, UV-B plus RF.

The expression ratio of pmTOR to mTOR was significantly higher in the skins of the UV-B group than those in the control and UV-B/RF groups (Figure 2A,B). In the UV-B/RF group, the ratio of pmTOR to mTOR was highest at 1 day after RF but also decreased with time (Figure 2A–C).

The expressions of autophagy initiation factors, such as FIP200, ULK1, ULK2, ATG13, and ATG101, were significantly lower in the UV-B group than in the control and UV-B/RF groups. In the UV-B/RF group, the expressions did not differ significantly among the time points after RF (Figure 2D–M).

### 2.3. RF Increased Melanosomal Autophagy and Degradation

**Figure 3:**
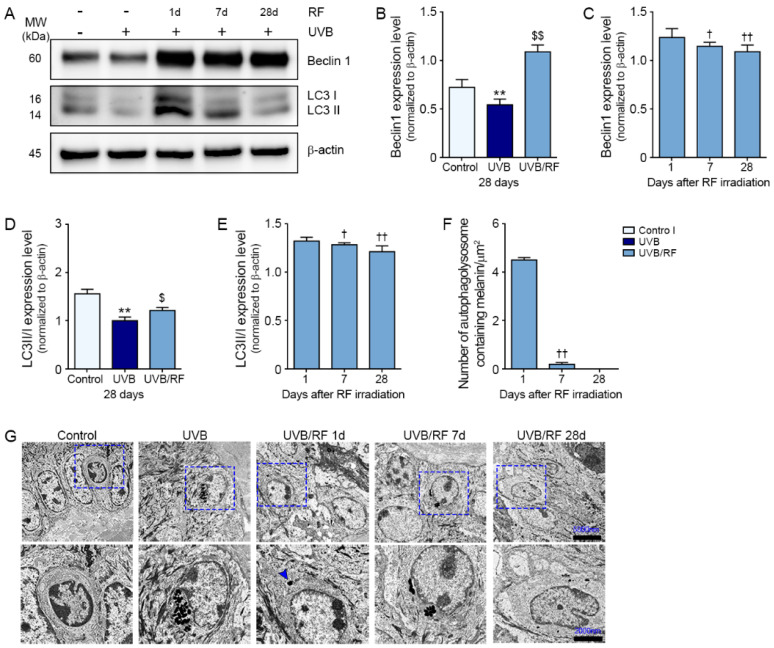
Activation of autophagosomes by RF irradiation in the UV-B-exposed animal model. (**A**) The expression levels of autophagy-related factors in skin tissue were assessed by Western blot; (**B**–**E**) Quantitative graphs of representative Western blot images. The protein expression levels were normalized to β-actin; (**F**) Quantitative graphs of representative TEM images; (**G**) Changes in autophagosome (arrows) numbers with time after RF irradiation were observed under TEM. Data are presented as mean ± SD. **, *p* < 0.01, vs. control (HEKn) or control 28 days (Skin); $, *p* < 0.05; $$, *p* < 0.01, vs. UV-B (HEKn) or UV-B 28 days (Skin); †, *p* < 0.05; ††, *p* < 0.01, vs. 1 day after RF irradiation (Mann–Whitney U test). LC3, microtubule-associated protein 1A/1B-light chain 3; RF, radiofrequency; TEM, transmission electron microscopy; UV-B, ultraviolet-B; UV-B/RF, UV-B plus RF.

Beclin 1 expression was significantly lower in the UV-B 28 days group than those in the control 28 days and UV-B/RF 28 days groups. In the UV-B/RF group, the expression of Beclin 1 was highest at 1 day after RF (Figure 3A–C).

The expression ratio of precursors of microtubule-associated protein light chain 3 (LC3)-like proteins to LC3-II in the UV-B group was significantly lower than that in the control 28 days group and the UV-B/RF 28 days group. In the UV-B/RF group, the expression ratio of LC3-I to LC3-II was highest at 1 day after RF (Figure 3A,D,E).

Melanosomal degradation by autophagy was evaluated by counting the number of melanin-containing autophagosomes in the transmission electron microscopy (TEM).

Melanin-containing autophagosomes were not observed in the control and UV-B-irradiated groups. Such autophagosomes observed from 1 day after RF irradiation disappeared at 28 days after RF irradiation. The number of melanin-containing autophagosomes was highest at 1 day after RF irradiation (Figure 3F,G). 

### 2.4. RF Decreased Skin Pigmentation

**Figure 4:**
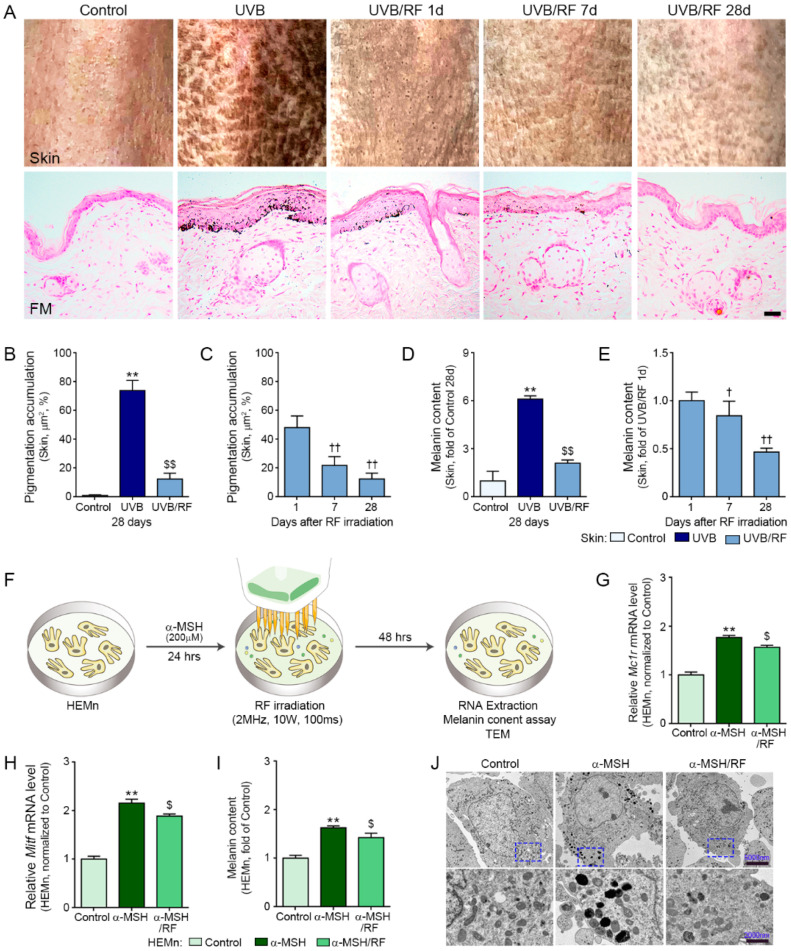
Reduction in melanin accumulation via autophagy activation by RF irradiation. (**A**) Pigmentation changes in UV-B-exposed mouse skin were assessed 1, 7, and 28 days after RF irradiation (upper row). Melanin content was assessed by FM staining (lower row; scale bar = 100 μm); (**B**,**C**) Quantitative graphs of representative mouse skin images and (**D**,**E**) representative FM staining images; (**F**) HEMn were treated either with α-MSH (200 μM) or with α-MSH/RF (2 MHz, 10 W for 100 ms) in vitro, or otherwise not irradiated (control); (**G**,**H**) The mRNA expression levels of MC1R (**F**), and MITF (**G**) were identified in the skin tissue. All mRNA levels were measured by qRT-PCR, normalized versus ACTB, and expressed relative to levels in the control; (**I**) Melanin content was measured in HEMn after RF irradiation; (**J**) Changes in melanin after RF irradiation were observed with TEM. Data are presented as mean ± SD. **, *p* < 0.01, vs. control 28 days (skin) or control (HEMn); $, *p* < 0.05; $$, *p* < 0.01, vs. UV-B 28 days (Skin) or α-MSH (HEMn); †, *p* < 0.05; ††, *p* < 0.01, vs. 1 day after RF irradiation (Mann–Whitney U test). α-MSH, alpha-melanotropin; FM, Fontana-Masson staining; HEMn, human epidermal melanocyte; NF-*κ*B, nuclear factor kappa-light-chain-enhancer of activated B cells; MC1R, melanocortin 1 receptor; MITF, micropthalmia-associated transcription factor; RF, radiofrequency; TEM, transmission electron microscopy; TNFR, tumor necrosis factor receptor; TLR4, Toll-like receptor 4; UV-B, ultraviolet-B; UV-B/RF, UV-B plus RF.

The areas of black spots in the skin of the UV-B group were significantly greater than those in the control and UV-B/RF groups 28 days after RF irradiation. In the UV-B/RF group, the areas of black spots were significantly largest at 1 day after RF (Figure 4A; upper panel, Figure 4C).

Fontana–Masson staining showed that melanin deposition in the UV-B group was significantly higher than those in the control and UV-B/RF groups. In the UV-B/RF group, melanin deposition was significantly highest at 1 day after RF (Figure 4A; lower panel, Figure 4D,E).

We also evaluated changes of melanin synthesis by RF with α-MSH-treated human epidermal melanocytes (HEMn) (F). The expression of MC1R and MITF were significantly increased by treating α-MSH, however those were significantly decreased by RF (Figure 4G,H). Melanin content that was evaluated with a melanin assay was significantly increased by treating α-MSH, however it was decreased by RF (Figure 4I). Images from TEM also showed that α-MSH increased melanin in the HEMn and melanin was decreased by RF (Figure 4J).

## 3. Discussion

Even though melanin protects the skin against UV radiation [23], excessive accumulation of melanin leads to hyperpigmentation-related disorders like melasma or freckles, which cause cosmetic problems [24]. Autophagy helps determine skin color by modulating melanosome degradation in keratinocytes [8]. Autophagic activity is related to the degree of skin pigmentation wherein keratinocytes in lighter skin are more able to degrading melanosomes than keratinocytes in darker skin [25]. A previous study showed that LC3B expression and autophagy were more decreased in melanocytes of melasma lesions than in unaffected areas of the skin [26]. NF-κB is activated by UV radiation [27]. The expressions of TNF-α and TLR4 are also increased by UV radiation [17,18,19,20], both of which downregulate autophagy by activating NF-κB [14,19,20]. In our study, we evaluated whether RF irradiation reduced skin pigmentation by restoring autophagy, which was decreased by UV-B radiation. We found that RF irradiation downregulated the expressions of TNFR, TLR4, and NF-κB, thereby increasing autophagic activity, an effect that was observed up to 28 days after RF irradiation.

Autophagy is initiated by the generation of double membrane-bound autophagosomes. In the autophagic process, autophagosomes form autolysosomes by merging with lysosomes [28]. Various stresses activate AMP-activated protein kinase (AMPK) and inhibit the mTOR, which consequently initiates autophagy by upregulating the FIP200, ULK 1, ATG13, and ATG101 [29]. After the initiation of autophagy, phagophore nucleation follows, which involves various proteins like ATG6 (Beclin 1), ATG14, and vacuolar protein sorting-associated protein 15 (Vps15) [29]. The next stage of nucleation is phagophore elongation. During elongation, precursors of LC3-like proteins are cleaved to produce LC3-II. By conjugation with phosphatidylethanolamine, the cytosolic form of LC-I becomes LC-II, which is an autophagosome-bound form [29]. LC3-II enhances targeted degradation of aggregated proteins or injured cellular organelles by interacting with adaptor proteins, such as p62 [10]. Thus, LC3-II is frequently used to measure autophagic flux [30].

Various cellular stress signals lead to the activation of mTORC1, which is an upstream target of the autophagy core machinery and the inactivation of which initiates autophagy [31]. mTORC1 inhibition activates ULK1/2 kinase activity, and then ULK1 and ULK2 phosphorylate ATG13 and FIP200, which are essential subunits of the ULK1/2 kinase complex [31,32,33]. In our study, pmTOR expression was increased by UV-B radiation and conversely decreased by RF radiation. Our findings suggest that RF irradiation promotes autophagy by inactivating mTOR, which otherwise inhibits autophagy and autophagic flux. We also evaluated whether RF irradiation contributed to melanosome removal by autophagy on TEM and found that an increase in the melanin-containing autophagosomes was observed from 1 day to 7 days after RF irradiation. Melanin deposition in the skin was increased by UV-B but decreased by RF radiation. Increased skin melanin accumulation is resulted from decreased melanin removal and increased melanin synthesis. Thus, we also evaluated whether changes of melanin synthesis by RF might also involve decreasing melanogenesis, and which possible mechanism might decrease inflammatory signal pathways such as TNF by RF. For evaluating exact mechanisms for decreasing melanogenesis by RF, further study is needed.

Various treatments have been suggested to reduce melanin accumulation, such as the use of hypopigmentation agents, which include tyrosinase inhibitors such as hydroquinone and arbutin, which block melanogenesis [34]. However, such agents are not very effective and cause irritation [34]. Natural products such as marliolide or ursolic acid have been found effective in reducing melanin deposition by increasing autophagy [34,35]. These studies showed that increasing autophagic activity led to melanin degradation and thus decreased skin pigmentation.

Our study showed that RF irradiation decreased skin pigmentation by increasing autophagy in skin in the UV-B-irradiated mouse model. RF irradiation decreased the expressions of TLR4 and TNFR, which in turn decreased mTOR activity, thereby increasing autophagic activity. Since we did not use knock-out animal models, it is hard to show which signal pathways were definitive ones to modulate autophagy by RF. To evaluate the exact mechanism of decreasing skin pigmentation by RF, future studies with knock-out animal models are needed. Moreover, this is a preclinical study which is too early to apply directly to humans.

Nevertheless, our results showed RF leads to increased autophagy which is associated with reduced melanin accumulation in the animal model. Our findings suggest that RF irradiation is a promising method of decreasing skin pigmentation by modulating autophagy.

## 4. Materials and Methods

### 4.1. In Vitro Model and RF Irradiation

Human primary epidermal keratinocytes (HEKn; American Type Culture Collection, ATCC, Manassas, VA, USA) were maintained with a keratinocyte growth kit (ATCC, Manassas, VA, USA). For establishing the in vitro model in HEKn, the cells were exposed to UV-B (200 mJ/cm^2^) for 5 min, irradiated with RF (POTENZA, Jeisys Medical Inc., Seoul, Korea; 2 MHz, 10 W, 100 ms), and incubated for 24 h (Figure 1A).

Human primary epidermal melanocytes (HEMn; ATCC, Manassas, VA, USA) were grown in Dermal Cell Basal Medium (ATCC, Manassas, VA, USA) with a melanocyte growth kit (ATCC, Manassas, VA, USA). For establishing the in vitro model in HEMn, the cells were treated with 200 nM α-MSH (Sigma Aldrich, St. Louis, MO, USA) and kept in an incubator at 37 °C in an atmosphere of 5% CO_2_ for 24 h. Then, the cells were irradiated with RF, and incubated for 48 h (Figure 4F).

### 4.2. Measurement of Melanin Content in Cells

To assess melanin content in HEMn, the cells were seeded at 1 × 10^4^ cells/well in 96-well plates and incubated for 24 h. After applying α-MSH and RF, the cells were harvested by centrifugation at 12,000× *g* for 20 min and dissolved in 100 μL of 10% dimethyl sulfoxide (DMSO) and 1N NaOH solution for 20 min at 95 °C. Absorbance at 490 nm was measured with a microplate reader (Molecular Devices).

### 4.3. In Vivo Model and RF Irradiation

Five-week-old male HRM-2 mice (20–25 g) were obtained from Central Lab Animal Inc. (Seoul, Korea) and cared to adapt for 2 weeks. The mice were housed in cages under a controlled temperature (23 °C) with a 12 h light/dark cycle and free access to food and water.

After the adaptation period, the mice were randomly divided into five groups as follows: (1) control (no exposure to UV-B and no irradiated RF), (2) UV-B (exposure to UV-B at 200 mJ/cm^2^), (3) UV-B/RF 1d (exposure to UV-B/irradiated RF; sampling proceeds 1 day after RF irradiation), (4) UV-B/RF 7d (exposure to UV-B/irradiated RF; sampling proceeds 7 days after RF irradiation), (5) UV-B/RF 28d (exposure to UV-B/irradiated RF; sampling proceeds 28 days after RF irradiation). The mice were exposed to UV-B for 5 min once every 2 days for 10 days and then for 5 min every day for the next 3 days (total of 13 days) [36]. Subsequently, the mice were irradiated to RF (2 MHz, 10 W for 100 ms) and then exposed to UV-B every 2 days for 28 days. The skin tissues of mice were harvested after 1 day, 7 days and 28 days of RF irradiation (Figure 1E). 

This study was approved by the Center of Animal Care and Use ethical board of Gachon University (Approval Number LCDI-2020-0115) and executed in accordance with the Institutional Animal Care and Use Committee. 

### 4.4. RF Irradiation System

The irradiation system (POTENZA, Jeisys Medical Inc., Seoul, Korea) used for this study was a bipolar pulse-type electrode array radiofrequency device. An impedance matching system was used to determine the compensation value by automatically measuring impedance, and RF was applied using a 16 ea (4 × 4) needle tip. RF was administered at 2 MHz using pulse-type, bipolar, alternating current oscillations in the animal experiment. Single pulse-type bipolar RF devices were used in the animal experiment and comprised an on-time pulse duration of 100 ms at a power density of 10 W/pulse. The invasive microneedle for RF application had a length of 13.6 mm, a diameter of 250 mm, and a needle-to-needle distance of 1.3 mm, and irradiation and treatment were performed with a disposable tip of 10 mm × 10mm consisting of 16 electrodes. The tip was approved by NAMSA (Northwood, OH, USA) after biological compatibility testing.

### 4.5. Sample Preparation

#### 4.5.1. Extraction of RNA and cDNA Synthesis

The cells and frozen skin tissues were ground using liquid nitrogen and homogenized by the RNAiso Plus reagent (Takara, Shiga, Japan) according to the manufacturer’s instructions.

The extracted RNA was quantified by the NanoDrop 2000 spectrophotometer (Thermo Fisher Scientific, Waltham, MA, USA) and was converted to cDNA using a PrimeScript 1^ST^ strand cDNA Synthesis Kit (Takara, Shiga, Japan) for quantitative real-time polymerase chain reaction (qRT-PCR).

#### 4.5.2. Paraffin-Embedded Tissue Sectioning

The skin tissues that were fixed by 4% paraformaldehyde (Sigma-Aldrich, St. Louis, MO, USA) were washed for 30 min for embedding. Skin paraffin blocks made using a tissue processor (Thermo Fisher Scientific, Waltham, MA, USA) were sectioned at 7-µm using a microtome (Leica, Wetzlar, Germany), and cooked at 37°C overnight to keep them attached to the slides. The sectioned slides were passed through xylene and four concentrations of ethanol (100%, 95%, 80%, and 70%) to deparaffinate them for staining.

#### 4.5.3. Isolation of Protein

The frozen skin tissues were ground using liquid nitrogen and homogenized by the RIPA buffer (EzRIPA, ATTO, Tokyo, Japan) with proteinase and phosphatase inhibitors. The homogenized skin tissues were sonicated and then centrifuged at 14,000× *g* for 15 min at 4 °C After centrifugation, the isolated protein (supernatant liquids) was aliquoted and quantified by a bicinchoninic acid assay kit (Thermo Fisher Scientific, Inc., Waltham, MA, USA).

### 4.6. Quantitative Real-Time Polymerase Chain Reaction

The qRT-PCR mixed a reagent containing the SYBR Green reagent (Takara), 1 µg of synthesized cDNA template, and a 10 pmol primer (Table S1), which were dispensed into 384-well multi-plates, and then analyzed by the CFX386 Touch Real-Time PCR System (Bio-Rad, Hercules, CA, USA). 

### 4.7. 3,3-Diaminobenzidine Staining for Immunohistochemistry Use

The sectioned skin tissue slides were incubated in 3% hydrogen peroxide in methanol for 30 min at room temperature to block endogenous peroxidase. The tissue slides were washed using a phosphate-buffered saline (PBS) and then incubated with mTOR antibodies (1:400; LSBio, Seattle, WA, USA) and pmTOR antibodies (1:50; Santa Cruz Biotechnology Inc., Dallas, TX, USA) in normal serum for 24 h at 4 °C. The slides were rinsed with PBS and incubated with a biotinylated secondary antibody using the ABC kit (Vector Laboratories Inc., Burlingame, CA, USA) for 2 h at room temperature. After washing with PBS, the tissue slides were developed using 3,3**′**-diaminobenzidine (Sigma-Aldrich) for 15 min to confirm the brown signal. To identify nuclei, tissue slides were stained in hematoxylin solution for 1 min, then mounted with dibutylphthalate polystyrene xylene mounting solution (Sigma-Aldrich). Images of the stained tissues were taken under an optical microscope (Olympus Optical Co., Tokyo, Japan) and analyzed using ImageJ software (NIH, Bethesda, MD, USA).

### 4.8. Western Blotting

Equal amounts of isolated skin proteins were separated on 8–12% polyacrylamide gels and transferred to polyvinylidene fluoride membranes (Millipore, Burlington, MA, USA) by a power station (ATTO, Osaka, Japan). After blocking using 5% skim milk and washing with Tris-buffered saline with 0.1% Tween 20 (TTBS), the membranes were incubated with Beclin 1 antibodies (1:2000; Bioss, Woburn, MA, USA), LC3 (1:500; Bioss) and β-actin (1:1000; Cell Signaling, Danvers, MA, USA) for 12 h at 4 °C and then washed with TTBS. The membranes were then incubated with a secondary antibody (Vector Laboratories, Burlingame, CA, USA) and rinsed with TTBS. Subsequently, an enhanced chemiluminescence detection reagent (GE Healthcare, Chicago, IL, USA) was used to visualize the immunoreactive proteins on the membrane.

### 4.9. Transmission Electron Microscopy

Specimens were fixed for 12 h in 2% glutaraldehyde/2% paraformaldehyde in 0.1 M phosphate buffer (pH 7.4) and washed in 0.1 M phosphate buffer, post-fixed with 1% OsO_4_ in 0.1 M phosphate buffer for 2 h, dehydrated with an ascending ethanol series (50%, 60%, 70%, 80%, 90%, 95%, 100%, and 100%) for 10 min each, and infiltrated with propylene oxide for 10 min.

The fixed samples were embedded using a Poly/Bed 812 kit (Polysciences, Warrington, PA, USA) and polymerized in an electron microscope oven (DOSAKA, Katsumi, Japan) at 65 °C for 12 h. The block was equipped with a diamond knife in the ultramicrotome, cut into 200 nm sections, and stained with toluidine blue for optical microscopy. 

The region of interest was then cut into 80 nm sections using the ultramicrotome, placed on copper grids, double stained with 3% uranyl acetate for 30 min and 3% lead citrate for 7 min, and observed under a TEM (JEOL, Tokyo, Japan) equipped with a Megaview III CCD camera (Soft Imaging System-Germany) at an acceleration voltage of 80 kV.

### 4.10. Fontana–Masson Staining

The skin tissues were incubated in Fontana ammoniacal silver solution (ScyTek, West Logan, UT, USA) overnight at room temperature, subsequently rinsed three times with distilled water, and then incubated in hypo solution for 3 min. Afterwards, the tissues were washed in distilled water, counterstained with neutral red stain for 5 min, washed in distilled water, dehydrated in absolute alcohol, and mounted for observation.

### 4.11. Statistical Analysis

We performed a Kruskal–Wallis test for comparisons of three groups, followed by a Mann–Whitney U test as a post hoc test. This study was validated using an unpaired *t*-test. All results are presented as mean ± standard deviation, and the statistical significance was displayed: *, vs. control (HEKn or HEMn) or control 28 days (skin); $, vs. UV-B (HEKn) or α-MSH (HEMn) or UV-B 28 days (skin); †, vs. UV-B/RF 1 day (skin). All statistical analyses were performed using SPSS version 22 (IBM Corporation; Armonk, NY, USA).

## Data Availability

All data is contained within the article.

## Supplementary Materials

The following are available online at https://www.mdpi.com/article/10.3390/ijms221910724/s1, Table S1: Primer list for quantitative polymerase chain reaction used in this study.
