# Supplementary material for: Evaluating Whether Radiofrequency Irradiation Attenuated UV-B-Induced Skin Pigmentation by Increasing Melanosomal Autophagy and Decreasing Melanin Synthesis
Source: Int J Mol Sci. 2021 Oct 3;22(19):10724. doi: 10.3390/ijms221910724 (PMC8509725; doi:10.3390/ijms221910724)
Supplement: Supplementary file 1 [file ijms-22-10724-s001.zip › ijms-1393774-supplementary.pdf]

International Journal of *Molecular Sciences*

MDPI

Supplementary Tables

Table S1. Primer list for quantitative polymerase chain reaction used in this study.

Gene	Species	Primers	
TNFR	Human	Forward	5′-GAA TGT TAA GGG CAC TGA GGA C-3′
		Reverse	5′-AGG AGG GAT AAA AGG CAA AGA C-3′
TLR4		Forward	5′-TTC TTG CTG GCT GCA TAA AGT A-3′
		Reverse	5′-CAT TCC TTA CCC AGT CCT CAT C-3′
NF-κB		Forward	5′-GGA CCA GCA AAG GTT ATT GTT C-3′
		Reverse	5′-AGA TCC CAT CCT CAC AGT GTT T-3′
MC1R		Forward	5'-TCT GGG TTC TCT CAA CTC CAA T-3′
		Reverse	5'-CAT CTG GGA TGG ACA CAT ACA G-3′
MITF		Forward	5'-ATC ATC AGC CTG GAA TCA AGT T-3′
		Reverse	5'-ATC AAG TTT CCA GAG ACG GGT A-3′
ACTB		Forward	5′-GGG ACC TGA CTG ACT ACC TCA T-3′
		Reverse	5′-CCT TAA TGT CAC GCA CGA TTT-3′
Tnfr	Mouse	Forward	5′-GAG TGA GAC ACA CTT CCA GTG C-3′
		Reverse	5′-ACA CGG TGT TCT GAG TCT CCT T-3′
Tlr4		Forward	5′-ATT CAG AGC CGT TGG TGT ATC T-3′
		Reverse	5′-TCA AGG ACA ATG AAG ATG ATG C-3′
Nf-κb		Forward	5′-AGA AAT CCT ACC CAC AGG TCA A-3′
		Reverse	5′-CAT TTG TGA CCA ACT GAA CGA T-3′
Fip200		Forward	5′-TGA ACA GAA AGA GCT TGC TCA G-3′
		Reverse	5′-GGT AAC ACA GAT GCA TCC TTC A-3′
Ulk1		Forward	5′-CCA GGA AAT GGC TAA TTC TGT C-3′
		Reverse	5′-GCA TAG TGT GCA GGT AGT CAG C-3′
Ulk2		Forward	5′-AAG GGA TAA TCC ACA GGG ATC T-3′
		Reverse	5′-ACC ACT GAC ATT CGA CTT CCT T-3′
Atg13		Forward	5′-CAT TTT CAG ACC CTA CTC CTG-3′
		Reverse	5′-CTT GAG AGT TGAT GGG AAA AGG-3′
Atg101		Forward	5′-ACT ACA AGA AGG AGG GCA CGT A-3′
		Reverse	5′-GCA CAT AGG TGA AGT CGA TGA A-3′
Actb		Forward	5'-CCG TAA AGA CCT CTA TGC CAA C-3′
		Reverse	5'-GCA GTA ATC TCC TTC TGC ATC C-3′
